# Control of Blood Glucose for Type-1 Diabetes by Using Reinforcement Learning with Feedforward Algorithm

**DOI:** 10.1155/2018/4091497

**Published:** 2018-12-30

**Authors:** Phuong D. Ngo, Susan Wei, Anna Holubová, Jan Muzik, Fred Godtliebsen

**Affiliations:** ^1^UiT The Arctic University of Norway, Tromsø, Norway; ^2^The University of Melbourne, Australia; ^3^Czech Technical University, Prague, Czech Republic

## Abstract

**Background:**

Type-1 diabetes is a condition caused by the lack of insulin hormone, which leads to an excessive increase in blood glucose level. The glucose kinetics process is difficult to control due to its complex and nonlinear nature and with state variables that are difficult to measure.

**Methods:**

This paper proposes a method for automatically calculating the basal and bolus insulin doses for patients with type-1 diabetes using reinforcement learning with feedforward controller. The algorithm is designed to keep the blood glucose stable and directly compensate for the external events such as food intake. Its performance was assessed using simulation on a blood glucose model. The usage of the Kalman filter with the controller was demonstrated to estimate unmeasurable state variables.

**Results:**

Comparison simulations between the proposed controller with the optimal reinforcement learning and the proportional-integral-derivative controller show that the proposed methodology has the best performance in regulating the fluctuation of the blood glucose. The proposed controller also improved the blood glucose responses and prevented hypoglycemia condition. Simulation of the control system in different uncertain conditions provided insights on how the inaccuracies of carbohydrate counting and meal-time reporting affect the performance of the control system.

**Conclusion:**

The proposed controller is an effective tool for reducing postmeal blood glucose rise and for countering the effects of external known events such as meal intake and maintaining blood glucose at a healthy level under uncertainties.

## 1. Introduction

Type-1 diabetes is a chronic condition that is characterized by an excessive increase in blood glucose level because the pancreas does not produce insulin hormone due to the autoimmune destruction of pancreatic beta cells. High blood glucose can lead to both acute and chronic complications and eventually result in failure of various organs.

Until today, there are many challenges in control of the blood glucose in type-1 diabetes. One of them is that the glucose kinetics process is complex, nonlinear, and only approximately known [1]. There are also many external known and unknown factors that affect the blood glucose level such as food intakes, physical activities, stress, and hormone changes. Generally, it is difficult to predict and quantify those factors and disturbances.

By using control theories, various studies have been conducted to design a control system for patients with type-1 diabetes. For example, Marchetti et al. [2], derived an improved proportional-integral-derivative controller for blood glucose control. Soylu et al. [3] proposed a Mamdani type fuzzy control strategy for exogenous insulin infusion. Model predictive control has also been widely used in type-1 diabetes and artificial pancreas development [4, 5]. Recently, together with the development of artificial intelligence and machine learning, reinforcement learning (RL) has emerged as a data-driven method to control unknown nonlinear systems [6, 7] and has been used as a long-term management tool for chronic diseases [8, 9]. The biggest advantage of RL compared to other methods is that the algorithm depends only on interactions with the system and does not require a well represented model of the environment. This especially makes RL well suited for type-1 diabetes since the modelling process of the insulin-kinetic dynamics is complex and requires invasive measurements on the patient or must be fit through a large dataset. Hence, by using RL as the control algorithm, the modelling process can be bypassed, which makes the algorithm not susceptible to any modelling error.

In diabetes, controlling of blood glucose require actions that are made at specific instance throughout the day in terms of insulin doses or food intakes. The actions are based on the current observable states of the patients (e.g., blood glucose measurement and heart rate). The effectiveness of the actions is calculated by how far the measured blood glucose value is compared to the healthy level. In RL, an agent makes decision based on the current state of the environment. The task of the algorithm is to maximize a cumulative reward function or to minimize a cumulative cost function. Based on these similarities in the decision-making process between a human being and a RL agent, RL may be key to the development of an artificial pancreas system.

When dealing with meal disturbances, modelling of glucose ingestion is the norm as well as the first step in designing a controller for disturbance rejection [10]. Feed-forward control was proven to be an effective tool to improve disturbance rejection performance [11, 12]. In control system theory, feed-forward is the term that describes a controller that utilizes the signal obtained when there is a (large) deviation from the model. Compared to feed-back control, where action is only taken after the output has moved away from the setpoint, the feed-forward architecture is more proactive since it uses the disturbance model to suggest the time and size of control action. Furthermore, building a meal disturbance model is simpler and requires less data to fit than finding the insulin-glucose kinetics. Based on the model, necessary changes in insulin actions can be calculated to compensate for the effects of carbohydrate on the blood glucose level.

A challenge in the control of the blood glucose is the lack of real-time measurement techniques. With the development of continuous glucose measurement sensors, blood glucose level can be measured and provided to the controller in minute intervals. However, blood glucose value alone is usually not enough to describe the states of the system for control purpose. Therefore, an observer is needed to estimate other variables in the state space from the blood glucose measurement. In this paper, the Kalman filter was chosen for that purpose since it can provide an optimal estimation of the state variables when the system is subjected to process and measurement noises [13, 14].

Vrabie et al. [15] established methodologies to obtain optimal adaptive control algorithms for dynamical systems with unknown mathematical models by using reinforcement learning. Based on that, Ngo et al. [16] proposed a reinforcement learning algorithm for updating basal rates in patients with type-1 diabetes. This paper completes the framework for blood glucose control with both basal and bolus insulin doses. The framework includes the reinforcement learning algorithm, the feed-forward controller for compensating food intake and the Kalman filter for estimating unmeasurable state variables during the control process. This paper also conducts simulations under uncertain information to evaluate the robustness of the proposed controller.

## 2. Methods

### 2.1. Problem Formulation

**Figure 1:**
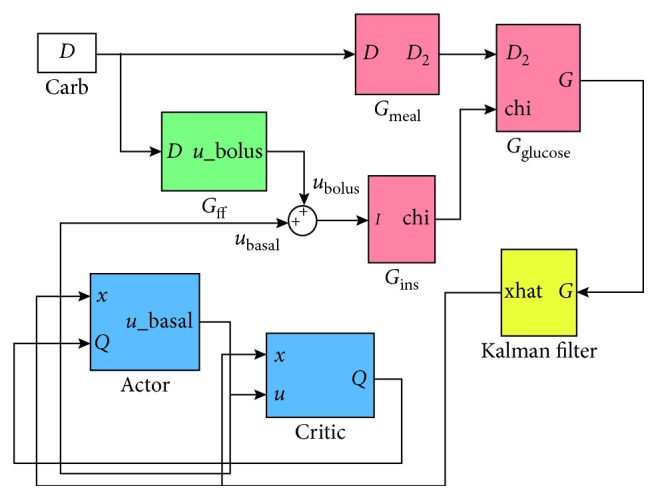
Control system diagram.

The purpose of our study is to design an algorithm to control the blood glucose in patients with type-1 diabetes by the means of changing the insulin concentration. The blood glucose metabolism is a dynamic system in which the blood glucose changing over time as the results of many factors such as food intake, insulin doses, physical activities, and stress level. The learning process of RL is based on the interaction between a decision-making agent and its environment, which will lead to an optimal action policy that results in desirable states [17]. The RL framework for type-1 diabetes includes the following elements:

- The state vector at time instance *k* consists of the states of the patient:

(1) $$\begin{array}{c} x_{k}={\left[\begin{array}{cc} g\left(k\right)-g_{d}\left(k\right) & \chi \left(k\right) \end{array}\right]}^{T}, \end{array}$$

where *g*(*k*) and *g*_d_(*k*) the are measured and desired blood glucose levels, respectively, and *χ*(*k*) is the interstitial insulin activity (defined in the appendix).

(ii) The control variable (insulin action) *u*_*k*_, which is part of the total insulin *i*_*k*_ (a combination of the basal and the bolus insulin (Figure 1)):

(2) $$\begin{array}{c} i_{k}=u_{\text{basal}}\left(k\right)+u_{\text{bolus}}\left(k\right)=u_{k}+u_{\text{basal}}\left(0\right)+u_{\text{bolus}}\left(k\right), \end{array}$$

where *u*_basal_(*k*) and *u*_bolus_(*k*) are the basal and bolus at time instance *k*, respectively.

(iii) The cost received one time-step later as a consequence of the action. In this paper, the cost was calculated by the following quadratic function:

(3) $$\begin{array}{c} r_{k+1}=x_{k}^{T}Qx_{k}+u_{k}^{T}Ru_{k}, \end{array}$$

where $Q=\left[\begin{array}{cc} 1 & 0\\ 0 & 0.1 \end{array}\right]$ and *R*=0.01. Each element in matrix **Q** and the value of *R* indicate the weighting factors of the cost function. The element in the first row and the first column of **Q** has the highest value, which corresponds to the weighting of the difference between the measured blood glucose and the prescribed healthy value. Since our ultimate goal is to reduce this difference, the factor of this measurement should have the largest value in the cost function. The element in the second row and second column of **Q** corresponds to the weighting of the interstitial insulin activity. The value of *R* indicates the weighting factor of the action (basal update). Minimizing the cost function, therefore, becomes the problem of minimizing the difference between the measured blood glucose and the desired value, the interstitial insulin activity, and the change in basal insulin.

At time instance *k*+1, a sequence of observations would be **x**_*k*_, *u*_*k*_, *r*_*k*+1_, **x**_*k*+1_ and *u*_*k*+1_. Based on this observation, the agent receives information about the state of the patient and chooses an insulin action. The body reacts to this action and transitions to a new state. This determines the cost of the action.

For the control design purpose, the blood glucose model (Appendix) was divided into three submodels: the meal (*G*_meal_), the insulin (*G*_ins_), and the glucose kinetics (*G*_glucose_). The controller has three main components: the actor, the critic, and the feedforward algorithm. The actor is used to estimate the action-value function, the critic's task is to obtain optimal basal insulin, and the feedforward algorithm is used to propose the bolus insulin profile for disturbance compensation (food intake). The purpose of the Kalman filter is to estimate unmeasurable states of the patient.

### 2.2. Basal Update by Actor and Critic

When the patient is in a fasting condition, the controller only needs to change the basal insulin level through the actor and the critic. Based on the current state **x**_*k*_, the actor proposes an insulin action *u*_*k*_ through the policy *π* : *u*_*k*_=*π*(**x**_*k*_). The updated basal rate is obtained from *u*_*k*_ as follows:

(4) $$\begin{array}{c} u_{\text{basal}}=u_{k}+u_{\text{basal}}\left(0\right). \end{array}$$

After each action, the patient transforms into a new state, and the cost associated with the previous action can be calculated using equation (3). The action-value function (*Q*-function) of action *u* is defined as the accumulation of cost when the controller takes action *u*_*k*_=*u* at time instance *k* and then continues following policy *π*(**x**_*k*+1_):

(5) $$\begin{array}{c} Q_{k}^{\pi }\left(x,u\right)=E_{\pi }\left\{\overset{\infty }{\underset{i=0}{\sum }}\gamma^{i}r_{k+i+1}\;|\;x_{k}=x,u_{k}=u\right\}, \end{array}$$

where *γ* (with 0 < *γ* ≤ 1) is the discount factor that indicates the weighting of future cost in the action-value function.

The action-value function depends on the current state and the next action. It was shown that the action-value function satisfies the following recursive equation (Bellman equation) [15, 17]:

(6) $$\begin{array}{c} Q_{k}^{\pi }\left(x,u\right)=r_{k}+\gamma Q_{k+1}^{\pi }\left(x,u\right). \end{array}$$

Since the state space and action space are infinite, function approximation was used in this paper for estimation of the *Q*-function. In this case, the *Q*-function was approximated as a quadratic function of vectors **x**_*k*_ and *u*_*k*_:

(7) $$\begin{array}{c} Q_{k}^{\pi }\left(x,u\right)\approx z_{k}^{T}Pz_{k}, \end{array}$$

where the symmetric and positive definite matrix **P** is called the kernel matrix and contains the parameters that need to be estimated. Vector **z**_*k*_ is the combined vector of **x**_*k*_ and *u*_*k*_:

(8) $$\begin{array}{c} z={\left[\begin{array}{cc} x_{k}^{T} & u_{k}^{T} \end{array}\right]}^{T}. \end{array}$$

With Kronecker operation, the approximated *Q*-function can be expressed as a linear combination of the basis function Φ(**z**_*k*_)=**z**_*k*_  ⊗  **z**_*k*_:

(9) $$\begin{array}{c} Q_{k}^{\pi }\left(x,u\right)\approx z_{k}^{T}Pz_{k}=w^{T}\left(z_{k}\;⊗\;z_{k}\right)=w^{T}\Phi \left(z_{k}\right), \end{array}$$

where **w** is the vector that contains elements of **P** and ⊗ is the Kronecker product.

By substituting *Q*_*k*_^*π*^(**x**, *u*) in equation (6) by **w** ^*T*^Φ(**z**_*k*_) and using the policy iteration method with the least square algorithm [15], elements of vector **w** can be estimated. Matrix **P** can then be obtained from **w** using the Kronecker transformation.

By decomposing the kernel matrix **P** into smaller matrices **P**_**xx**_, **P**_**x***u*_, **P**_*u ***x**_, and **P**_*uu*_, the approximated *Q*-function can be written as follows:

(10) $$\begin{array}{c} Q_{k}^{\pi }\left(x,u\right)=\frac{1}{2}{\left[\begin{array}{c} x_{k}\\ u_{k} \end{array}\right]}^{T}P\left[\begin{array}{c} x_{k}\\ u_{k} \end{array}\right]=\frac{1}{2}{\left[\begin{array}{c} x_{k}\\ u_{k} \end{array}\right]}^{T}\left[\begin{array}{cc} P_{xx} & P_{xu}\\ P_{ux} & P_{uu} \end{array}\right]\left[\begin{array}{c} x_{k}\\ u_{k} \end{array}\right]. \end{array}$$

The current policy is improved with actions that minimize the *Q*-function *Q*_*k*_^*π*^(**x**, *u*). This can be done by first taking the partial derivative of the *Q*-function and then solving ∂*Q*_*k*_^*π*^(**x**, *u*)/∂*u*=0. The optimal solution can thereafter be obtained as follows [15]:

(11) $$\begin{array}{c} u_{k}=-P_{uu}^{-1}P_{ux}x_{k}. \end{array}$$

With that, the update of basal insulin is

(12) $$\begin{array}{c} u_{\text{basal}}=-P_{uu}^{-1}P_{ux}x_{k}+i_{\text{be}}, \end{array}$$

where *i*_be_ is the equilibrium basal plasma insulin concentration.

### 2.3. Bolus Update by Feedforward Algorithm

When the patient consumes meals, in addition to the basal insulin, the controller calculates and applies boluses to compensate for the rise of blood glucose as the results of carbohydrate in the food. The feedforward algorithm first predicts how much blood glucose level will rise and then suggests a bolus profile to counter the effects of the meal. The starting time of the bolus doses was also calculated by the algorithm based on the meal intake model.

Since the meal intake model (equations (A.1) and (A.2)) and the insulin model (equation (A.4)) are linear time-invariant (LTI) models, they can be transformed from state space equations into transfer functions as follows:

(13) $$\begin{array}{c} G_{\text{meal}}\left(s\right)=\frac{D_{2}\left(s\right)}{D\left(s\right)}=C_{\text{meal}}{\left(sI-A_{\text{meal}}\right)}^{-1}B_{\text{meal}}=\frac{A_{G}}{{\left(s\tau_{D}+1\right)}^{2}},\\ G_{\text{ins}}\left(s\right)=\frac{D_{2}\left(s\right)}{D\left(s\right)}=C_{\text{ins}}{\left(sI-A_{\text{ins}}\right)}^{-1}B_{\text{ins}}=\frac{p_{3}}{s+p_{2}}, \end{array}$$

where

(14) $$\begin{array}{c} A_{\text{meal}}=\left[\begin{array}{cc} -1/\tau_{D} & 0\\ 1/\tau_{D} & -1/\tau_{D} \end{array}\right],\\ B_{\text{meal}}=\left[\begin{array}{c} A_{G}\\ 0 \end{array}\right],\\ C_{\text{meal}}=\left[\begin{array}{cc} 0 & 1/\tau_{D} \end{array}\right],\\ A_{\text{ins}}=-p_{2},\\ B_{\text{ins}}=p_{3},\\ C_{\text{ins}}=1. \end{array}$$

Descriptions and values of *τ*_*D*_, *p*_2_, and *p*_3_ are shown in Tables 1 and 2. The transfer function from the meal intake *D*(*s*) to the blood glucose level *g*(*s*) can be calculated as

(15) $$\begin{array}{c} F\left(s\right)=\frac{g\left(s\right)}{D\left(s\right)}=\left(G_{\text{meal}}\left(s\right)+G_{\text{ff}}\left(s\right)G_{\text{ins}}\left(s\right)\right)G_{\text{glucose}}\left(s\right). \end{array}$$

In order to compensate for the meal, the gain of the open loop system *F*(*s*) must be made as small as possible. Hence, the feedforward transfer function was chosen such that *G*_meal_(*s*)+*G*_ff_(*s*)*G*_ins_(*s*)⟶0, which leads to

(16) $$\begin{array}{c} G_{\text{ff}}\left(s\right)=-G_{\text{meal}}\left(s\right)G_{\text{ins}}^{-1}\left(s\right)=\frac{-A_{G}\left(s+p_{2}\right)}{p_{3}{\left(\tau_{D}s+1\right)}^{2}}. \end{array}$$

The meal compensation bolus in *s*-domain can be calculated from the feedforward transfer function:

(17) $$\begin{array}{c} u_{\text{bolus}}\left(s\right)=G_{\text{ff}}\left(s\right)D\left(s\right)=\frac{-A_{G}\left(s+p_{2}\right)}{p_{3}{\left(\tau_{D}s+1\right)}^{2}}D\left(s\right). \end{array}$$

Hence, the feedforward action becomes the output of the following dynamic system, which can be solved easily using any ordinary differential equation solver:

(18) $$\begin{array}{c} p_{3}\tau_{D}^{2}{\ddot{u}}_{\text{bolus}}\left(t\right)+2p_{3}\tau_{D}{\dot{u}}_{\text{bolus}}\left(t\right)+p_{3}u_{\text{bolus}}\left(t\right)=-A_{G}\left(\dot{D}\left(t\right)+p_{2}D\left(t\right)\right). \end{array}$$

### 2.4. Kalman Filter for Type-1 Diabetes System

Since the interstitial insulin activity, the amounts of glucose in compartments 1 and 2 cannot be measured directly during implementation, Kalman filter was used to provide an estimation of the state variables from the blood glucose level. The discretized version of the type-1 diabetes system can be written in the following form:

(19) $$\begin{array}{c} x_{K}\left(k+1\right)=A_{K}x_{K}\left(k\right)+B_{K}u_{K}\left(k\right)+H_{K}w\left(k\right),\\ y_{K}\left(k\right)=C_{K}x_{K}\left(k\right)+v\left(k\right), \end{array}$$

where $x_{K}\left(k\right)={\left[\begin{array}{cccc} D_{1} & D_{2} & g\left(k\right)-g_{d}\left(k\right) & \chi \left(k\right) \end{array}\right]}^{T}$, $u_{K}\left(k\right)={\left[\begin{array}{cc} D\left(k\right) & i\left(k\right) \end{array}\right]}^{T}$, and matrices **A**_*K*_, **B**_*K*_, **C**_*K*_ are linearized coefficient matrices of the model:

(20) $$\begin{array}{c} A_{K}=\left[\begin{array}{cccc} -1/\tau_{D} & 0 & 0 & 0\\ 1/\tau_{D} & -1/\tau_{D} & 0 & 0\\ 0 & 1/\tau_{D} & -p_{1}-g_{d} & 0\\ 0 & 0 & 0 & -p_{2} \end{array}\right],\\ B_{K}=\left[\begin{array}{cc} A_{G} & 0\\ 0 & 0\\ 0 & 0\\ 0 & p_{3}V \end{array}\right],\\ C_{K}=\left[\begin{array}{cccc} 0 & 0 & 1 & 0 \end{array}\right], \end{array}$$

matrix **H**_*K*_ is the noise input matrix: $H_{K}={\left[\begin{array}{cccc} 0 & 0 & 0 & p_{3}V \end{array}\right]}^{T}$, the output value *y*_*K*_(*k*)=*g*(*k*) − *g*_d_(*k*) is the measured blood glucose deviation from the desired level, *w*(*k*) is the insulin input noise, and *v*(*k*) is the blood glucose measurement noise with zero-mean Gaussian distribution. The variances of *w*(*k*) and *v*(*k*) are assumed to be as follows:

(21) $$\begin{array}{c} E\left(w^{2}\left(k\right)\right)=R_{w},\\ E\left(v^{2}\left(k\right)\right)=R_{v}. \end{array}$$

Based on the discretized model, a Kalman filter was implemented through the following equation:

(22) $$\begin{array}{c} \hat{x}\left(k+1\;|\;k\right)=A_{k}\cdot \hat{x}\left(k\;|\;k-1\right)+B_{k}\cdot u_{K}\left(k\right)+L\left[y\left(k\right)-C\cdot \hat{x}\left(k\;|\;k-1\right)\right], \end{array}$$

where $\hat{x}\left(k+1|k\right)$ denotes the estimation of **x**(*k*+1) based on measurements available at time *k*. The gain **L** is the steady-state Kalman filter gain, which can be calculated by

(23) $$\begin{array}{c} L=MC^{T}{\left(CMC^{T}+R_{v}\right)}^{-1}, \end{array}$$

where **M** is the solution of the corresponding algebraic Riccati equation [13, 14, 18]:

(24) $$\begin{array}{c} M=AMA^{T}+BR_{w}B-AMC^{T}{\left(CMC^{T}+R\right)}^{-1}CMA^{T}. \end{array}$$

By assuming the noise variances to be *R*_*w*_=*R*_*v*_=0.01, the Kalman filter gain was calculated from equation (23) as

(25) $$\begin{array}{c} L=\left[\begin{array}{cccc} 0 & 0 & 8.32\cdot 10^{-4} & -6.40\cdot 10^{-7} \end{array}\right]. \end{array}$$

### 2.5. Simulation Setup

First, a pretraining of the algorithm was conducted on the type-1 diabetes model in the scenario where the patient is in a fasting condition (without food intake). The purpose of the pretraining simulation is to obtain an initial estimation of the action-value function for the algorithm. The learning process was conducted by repeating the experiment multiple times (episodes). Each episode starts with an initial blood glucose of 90 mg/dL and ends after 30 minutes. The objective of the algorithm is to search and explore actions that can drive the blood glucose to its target level of 80 mg/dL.

By using the initial estimation of the action-value function, the controller was then tested in the daily scenario with food intake. Comparisons were made between the proposed reinforcement learning with the feedforward (RLFF) controller, the optimal RL (ORL) controller [15], and the proportional-integral-derivative (PID) controller. The ORL was designed with the same parameters and pretrained in the same scenario as with the RLFF. The PID controller gains were chosen, which produces a similar blood glucose settling time as the RLFF:

(26) $$\begin{array}{c} u_{k}=K_{p}\left(g\left(k\right)-g_{d}\left(k\right)\right)+K_{i}\underset{k}{\sum }\left(g\left(k\right)-g_{d}\left(k\right)\right)+K_{d}\left(g\left(k\right)-g\left(k-1\right)\right), \end{array}$$

where

(27) $$\begin{array}{c} K_{p}=1,\\ K_{i}=0.001,\\ K_{d}=0.01. \end{array}$$

**Figure 2:**
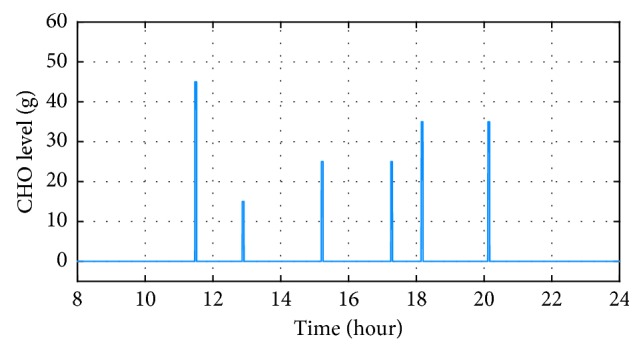
CHO consumed throughout the day.

In order to understand the effects of different food types on the controlled system, two sets of simulations were conducted for food that has slow and fast glucose absorption rates while containing a similar amount of carbs. Absorption rates in the simulations are characterized by parameter *τ*_*D*_ from the model, where *τ*_*D*_=50 corresponds to food with a slow absorption rate and *τ*_*D*_=10 corresponds to food with a fast absorption rate. The amount of carbohydrate (CHO) per meal can be found in Figure 2.

Next, the performance of the proposed controller was evaluated under uncertainties of meal information. Two cases of uncertainties were considered: uncertain CHO estimation case and uncertain meal-recording time. In the uncertain CHO estimation, the estimated CHO information that provided to the controller was assumed to be a normal distribution with a standard deviation of 46% from the correct carbohydrate value shown in Figure 2. The standard deviation value was used based on the average adult estimates and the computerized evaluations by the dietitian [19]. For the uncertain meal-recording time, the estimated meal starting time is assumed to be a normal distribution with a standard deviation of two minutes from the real starting time. This standard deviation value was randomly selected because systematic research on the accuracy of meal-time recording for patients with type-1 diabetes could not be found. For each case, multiple simulations were conducted in the same closed-loop system with its corresponding random variables. From the obtained results, the mean and standard deviation for blood glucose responses at each time point will be calculated and analyzed.

## 3. Results

After pretraining in the no-meal scenario, the *Q*-function was estimated as follows:

(28) $$\begin{array}{c} Q_{k}^{\pi }\left(x,u\right)=\left[\begin{array}{cc} x_{k}^{T} & u_{k}^{T} \end{array}\right]\left[\begin{array}{ccc} 4.454\cdot 10^{2} & -8.870\cdot 10^{4} & -0.084\\ -8.870\cdot 10^{4} & 3.538\cdot 10^{7} & 33.630\\ -0.084 & 33.630 & 0.010 \end{array}\right]\left[\begin{array}{c} x_{k}\\ u_{k} \end{array}\right]. \end{array}$$

The initial basal policy was obtained from the initial *Q*-function and equation (12):

(29) $$\begin{array}{c} u_{\text{basal}}\left(k\right)=8.86\left(g\left(k\right)-80\right)-3534.11\chi \left(k\right)+7.326. \end{array}$$

The initial estimation of the *Q*-function and the initial basal policy were used for subsequent testing simulations of the control algorithm.

**Figure 3:**
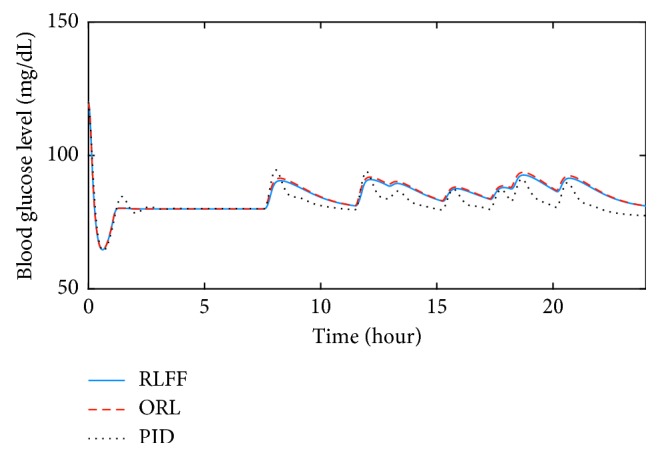
Comparison of the blood glucose responses in the nominal condition for slow glucose absorption food.

**Figure 4:**
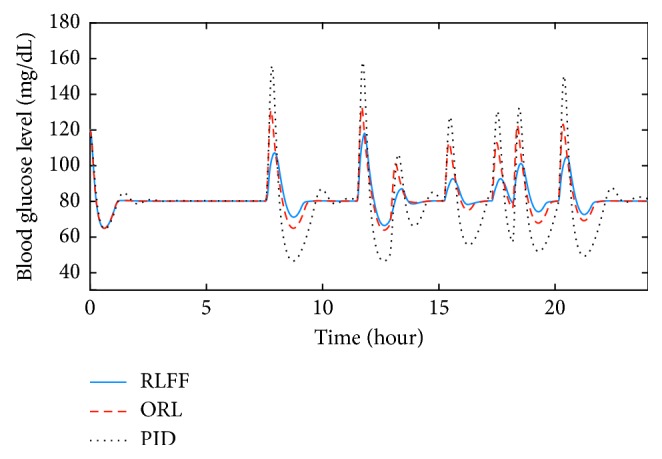
Comparison of the blood glucose responses in the nominal condition for fast glucose absorption food.

**Figure 5:**
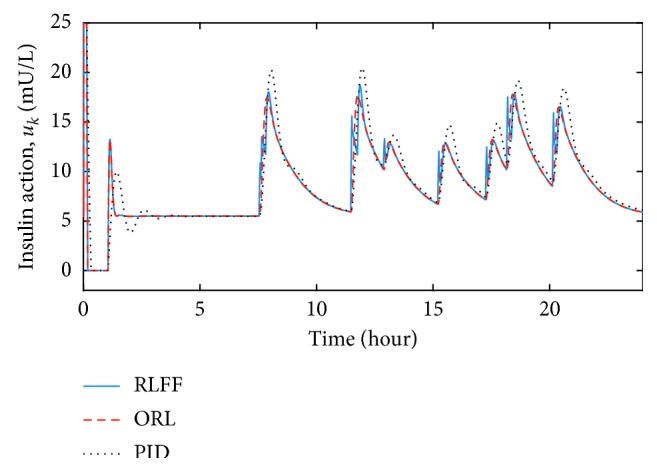
Comparison of insulin concentrations in the nominal condition for slow glucose absorption food.

**Figure 6:**
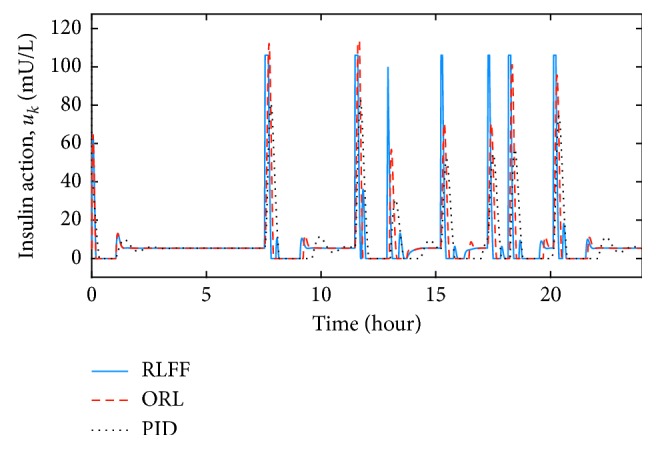
Comparison of insulin concentrations in the nominal condition for fast glucose absorption food.

During the simulation with correct meal information, blood glucose responses of the RLFF, the ORL, and the PID are shown in Figures 3 and 4. The insulin concentration during the process can also be found in Figures 5 and 6. With slow-absorption food, the fluctuation range of blood glucose was approximately ±30 mg/dL for all three controllers from the desired value (Figure 3). However, with fast absorption glucose meals, the fluctuation range of the postmeal blood glucose level was within ±40 mg/dL with the RLFF compared to ±60 mg/dL with the ORL and is significantly smaller than the fluctuation range ±80 mg/dL of the PID (Figure 4).

**Figure 7:**
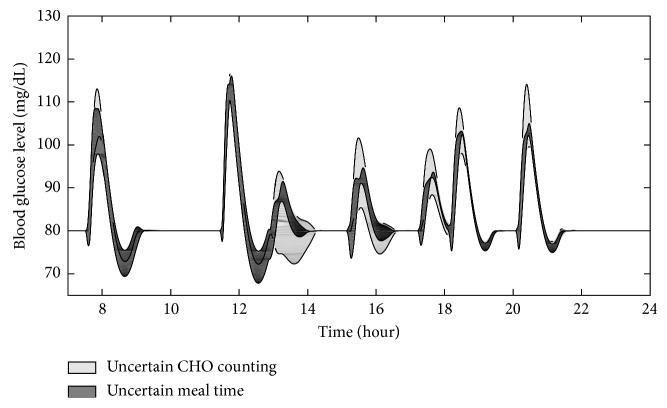
Blood glucose responses under uncertainties for fast glucose absorption food.

**Figure 8:**
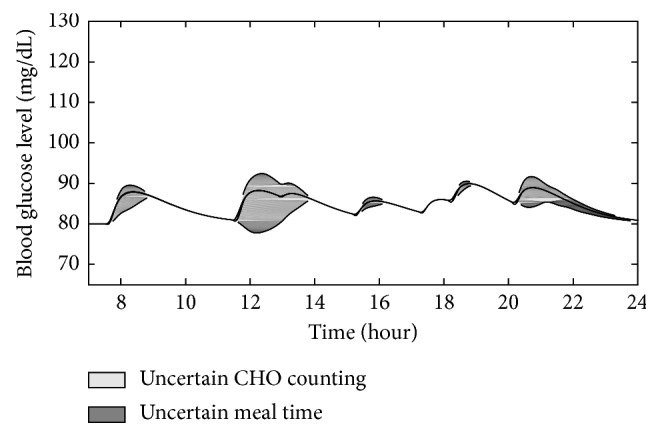
Blood glucose responses under uncertainties for slow glucose absorption food.

Figures 7 and 8 show the blood glucose variation under uncertain meal time and CHO counting. The upper and lower bounds in shaded areas show the mean blood glucose value plus and minus the standard deviation for each instance. Under uncertain meal information, the upper bound was kept to be smaller than 40 mg/dL from the desired blood glucose value for fast glucose absorption food and 15 mg/dL for slow glucose absorption food. The lower bound is smaller than 15 mg/dL from the desired value for fast glucose absorption food and 5 mg/dL for slow glucose absorption food.

## 4. Discussion

The controller has shown its capability to reduce the rise of postmeal blood glucose in our simulations. It can be seen in Figures 3 and 4 that three controllers were able to stabilize the blood glucose. However, when using the RLFF, the added bolus makes the insulin responses much faster when there is a change in blood glucose level, which reduces the peak of the postmeal glucose rise by approximately 30 percent compared to the ORL and 50 percent compared to the PID in the fast-absorption case. It can also be seen that the undershoot blood glucose (the distance between the lowest blood glucose and the desired blood glucose value) of the PID controller is much larger than that of the RLFF and the ORL. The RLFF has the smallest glucose undershoot among the three controllers. Low blood glucose value (hypoglycemia) can be very dangerous for patients with type-1 diabetes. Therefore, simulation results show the advantage of using RLFF in improving safety for patients. In general, with the feedforward algorithm, the proposed algorithm is an effective tool for countering the effects of external events such as meal intake.

Among uncertainties, carb counting created more effect on the variation of the blood glucose than meal-time recording, especially with slow absorbing food. The uncertainty in recording meal time may also lead to larger undershot of blood glucose below the desired level as can be seen in Figure 7. Following the same trend as previous simulations, the fluctuation range of the blood glucose with slow absorbing food is smaller than the fluctuation range with fast glucose absorbing food. In general, the control algorithm kept the blood glucose at the healthy level although uncertainties affect the variation of the responses. However, an accurate carbohydrate counting and accurate meal-time recording method are still important for the purpose of blood glucose control in order to completely avoid the chance of getting hypoglycemia.

## 5. Conclusion

The paper proposes a blood glucose controller based on reinforcement learning and feedforward algorithm for type-1 diabetes. The controller regulates the patient's glucose level using both basal and bolus insulin. Simulation results of the proposed controller, the optimal reinforcement learning, and the PID controller on a type-1 diabetes model show that the proposed algorithm is the most effective algorithm. The basal updates can stabilize the blood glucose, and the bolus can reduce the glucose undershoot and prevent hypoglycemia. Comparison of the blood glucose variation under different uncertainties provides understandings of how the accuracy of carbohydrate estimation and meal-recording time can affect the closed-loop responses. The results show that the control algorithm was able to keep the blood glucose at a healthy level although uncertainties create variations in the blood glucose responses.

## Figures and Tables

**Table 1:** Parameters and constants of the insulin-glucose kinetics model.

Name	Description	Value
*p*_1_	Glucose effectiveness	0.2 min^−1^
*p*_2_	Insulin sensitivity	0.028 min^−1^
*p*_3_	Insulin rate of clearance	10^−4^ min^−1^
*A*_*G*_	Carbohydrate bioavailability	0.8 min^−1^
*τ*_*D*_	Glucose absorption constant	10 min
*V*	Plasma volume	2730 g
*i*_be_	Equilibrium basal plasma insulin concentration	7.326 *μ*IU/ml

**Table 2:** Variables of the insulin-glucose kinetics model.

Name	Description	Unit
*D*	Amount of CHO intake	mmol/min
*D*_1_	Amount of glucose in compartment 1	mmol
*D*_2_	Amount of glucose in compartment 2	mmol
*g*(*t*)	Plasma glucose concentration	mmol/l
*χ*(*t*)	Interstitial insulin activity	min^−1^
*i*(*t*)	Plasma insulin concentration	*μ*IU/ml

## Data Availability

The data used to support the findings of this study are available from the corresponding author upon request.

## Appendix

### Blood Glucose Model

In this paper, the insulin-glucose process was used as the subject in our simulations. The model is described by the following equations [20–23]:

(A.1) $$\begin{array}{c} \frac{dD_{1}\left(t\right)}{dt}=A_{G}D\left(t\right)-\frac{D_{1}\left(t\right)}{\tau_{D}}, \end{array}$$

(A.2) $$\begin{array}{c} \frac{dD_{2}\left(t\right)}{dt}=\frac{D_{1}\left(t\right)}{\tau_{D}}-\frac{D_{2}\left(t\right)}{\tau_{D}}, \end{array}$$

(A.3) $$\begin{array}{c} \frac{dg\left(t\right)}{dt}=-p_{1}g\left(t\right)-\chi \left(t\right)g\left(t\right)+\frac{D_{2}\left(t\right)}{\tau_{D}}, \end{array}$$

(A.4) $$\begin{array}{c} \frac{d\chi \left(t\right)}{dt}=-p_{2}\chi \left(t\right)+p_{3}V\left(i\left(t\right)-u_{\text{be}}\right), \end{array}$$

where variable descriptions and parameter values are given in Tables 1 and 2. In this model, the inputs are the amount of CHO intake *D* and the insulin concentration *i*(*t*). The output of the model is the blood glucose concentration *g*(*t*). It is assumed that the blood glucose is controlled by using an insulin pump, and there is no delay between the administered insulin and the plasma insulin concentration.

## Abbreviations

RL: Reinforcement learning
RLFF: Reinforcement learning with feedforward algorithm
ORL: Optimal reinforcement learning
PID: Proportional-integral-derivative
LTI: Linear time-invariant
CHO: Carbohydrate.
